# The impact of transactive memory systems on innovation performance in undergraduate teams

**DOI:** 10.3389/fpsyg.2026.1720641

**Published:** 2026-04-16

**Authors:** Peng Hu, Die Hu, Maoyan She, Zufeng Li

**Affiliations:** 1 College of Management Science, Chengdu University of Technology, Chengdu, Sichuan, China; 2 Business School of Sichuan University, Sichuan University, Chengdu, Sichuan, China; 3 National Research Base of Intelligent Manufacturing Service, Chongqing Technology and Business University, Chongqing, China

**Keywords:** coordination, credibility, innovation performance, specialization, transactive memory system, undergraduate innovation teams

## Abstract

**Background:**

Transactive Memory Systems (TMS) are widely recognized as an important mechanism for knowledge integration and collaborative performance in teams. However, limited research has examined how different TMS dimensions operate jointly in temporary undergraduate innovation teams, which are typically characterized by limited formal structure, short interaction histories, and strong dependence on peer-based collaboration. To address this gap, this study investigates how specialization, credibility, and coordination influence innovation performance in undergraduate innovation teams, and further examines whether credibility and coordination strengthen the effect of specialization on innovation performance.

**Methods:**

Grounded in the knowledge integration perspective, this study develops a conceptual model of TMS and innovation performance. Using survey data collected from undergraduate teams participating in innovation and entrepreneurship programs, structural equation modeling (SEM) was employed to examine the relationships among specialization, credibility, coordination, and innovation performance, as well as the moderating effects of credibility and coordination on the relationship between specialization and innovation performance.

**Results:**

The findings show that specialization, credibility, and coordination each have a significant positive effect on innovation performance. Moreover, the impact of specialization on innovation performance is strengthened when teams exhibit higher levels of credibility and coordination.

**Originality/value:**

This study extends TMS research to undergraduate innovation teams and shows that TMS dimensions should be understood not only as distinct components, but also as interacting elements. It further contributes to innovation and entrepreneurship education research by explaining how interdisciplinary student teams can better translate distributed expertise into innovation outcomes through stronger peer credibility and collaborative coordination.

## 1 Introduction

Innovation is increasingly recognized as a collaborative process that depends on the sharing and integration of diverse knowledge and skills among team members (Ben-Menahem et al., 2016; van Knippenberg, 2017). In higher education, undergraduate innovation teams, such as those formed under national innovation and entrepreneurship education programs, play a crucial role in helping students develop creativity, problem-solving skills, and the ability to tackle real-world challenges (Wang and Horta, 2025). However, despite their educational value, such teams often face considerable difficulties: members usually come from different academic backgrounds, possess varied levels of expertise, and often lack systematic training in project and innovation management (Gray et al., 2023). Although such diversity can potentially stimulate creativity, it may also make it difficult for teams to organize, coordinate, and integrate distributed knowledge effectively. Therefore, how these challenges are managed largely determines team innovation performance and the overall effectiveness of innovation training in universities.

While prior research has emphasized the positive role of diversity and collaboration in driving innovation (Wen et al., 2021; Baruah et al., 2023), relatively few studies have examined the micro-level mechanisms that enable effective knowledge integration within non-professional small teams, especially those composed of undergraduate students. Compared with professional R&D teams, undergraduate innovation teams typically lack formal organizational structures, stable role arrangements, and established coordination routines, and their innovation processes rely more heavily on interpersonal trust, spontaneous collaboration, and shared cognitive coordination (Walden et al., 2015). This makes the cognitive and social dynamics within such teams especially relevant for understanding their innovation outcomes.

A promising theoretical lens for capturing such dynamics is the Transactive Memory System (TMS), which describes how teams collectively encode, store, retrieve, and communicate knowledge (Asim Shahzad et al., 2022; Kush et al., 2024). TMS represents a team’s internal system for knowledge coordination, and is commonly conceptualized as comprising three dimensions: specialization, credibility, and coordination. Specialization refers to members’ differentiated knowledge domains and shared awareness of who knows what; credibility reflects the extent to which members trust one another’s expertise; and coordination captures the team’s ability to work together smoothly and efficiently. Together, these dimensions reflect the cognitive and social foundations of collective knowledge processing (Yan et al., 2021).

Although TMS has often been treated as an overall team-level construct, a growing number of studies have begun to examine its dimensions separately in innovation-related outcomes. For example, Wang et al. (2025) showed that specialization, credibility, and coordination exert distinct effects on breakthrough innovation in inventor teams and further demonstrated that these effects vary with technological complexity and team size. Utami et al. (2024), in contrast, examined TMS in inter-organizational collaboration and argued that specialization provides a structural basis for distributed knowledge, whereas trust and coordination help transform that knowledge structure into innovation and performance outcomes. These studies have advanced the literature by showing that TMS dimensions are not functionally identical and, in some settings, may complement one another. However, existing dimensional-level TMS research has mainly focused on professional R&D teams or inter-organizational collaborations, where members often benefit from clearer role structures, stronger task routines, and more formalized collaboration mechanisms. By contrast, much less is known about how TMS operates in temporary undergraduate innovation teams, which are typically weakly formalized, short-lived, and highly dependent on peer interaction. More importantly, prior studies have mainly emphasized either the differentiated effects of TMS dimensions or specialization-centered structure–process logic, while paying less attention to how social-interaction dimensions (credibility and coordination) may shape the effectiveness of the cognitive dimension (specialization) within temporary teams.

This gap is theoretically important because the functioning of TMS in temporary teams may depend not only on whether its dimensions are distinguishable, but also on how they work together. In undergraduate innovation teams, specialization reflects the cognitive map of distributed expertise, yet such expertise is unlikely to generate innovation value unless members trust one another’s knowledge and can coordinate their contributions efficiently. In other words, the benefits of specialization may be contingent on the presence of credibility and coordination. In this sense, credibility and coordination do not merely coexist with specialization as parallel dimensions, they may also influence whether and how the cognitive value of specialization is realized in practice.

Against this background, this study investigates how TMS influences innovation performance in undergraduate innovation teams. Specifically, rather than conceptualizing TMS only as a composite construct, we examine the distinct effects of specialization, credibility, and coordination on innovation performance, and further test whether credibility and coordination moderate the relationship between specialization and innovation performance. In doing so, we focus not only on the differentiated functions of TMS dimensions, but also on their interrelationships in explaining team innovation performance.

Using survey data from undergraduate teams participating in innovation and entrepreneurship programs in Chinese universities, we empirically examine (1) the effects of the three TMS dimensions on innovation performance and (2) the moderating effects of credibility and coordination on the relationship between specialization and innovation performance. This study makes three contributions. First, it extends dimensional-level TMS research to temporary undergraduate innovation teams, a form of collaboration characterized by limited formal structure, short interaction histories, and strong dependence on peer-based knowledge integration. Second, it advances the broader TMS literature by showing that the three dimensions of TMS should be understood not only as distinct components, but also as interacting elements, especially in the sense that social-interaction dimensions such as credibility and coordination can shape the extent to which the cognitive value of specialization is translated into innovation performance. Third, it contributes to research on innovation and entrepreneurship education by explaining how interdisciplinary student teams can better transform distributed expertise into innovation outcomes through trust-building and coordination-enhancing practices. These findings also provide practical implications for instructors, mentors, and program designers seeking to foster more effective student innovation teams through interventions that strengthen both peer trust and collaborative coordination.

## 2 Literature review

### 2.1 TMS in team contexts

The concept of the TMS describes how members of a group collectively encode, store, and retrieve information through a shared understanding of “who knows what.” TMS captures both the cognitive and social mechanisms that enable teams to process distributed knowledge effectively (Asim Shahzad et al., 2022). Over time, scholars have conceptualized TMS as comprising three interrelated dimensions: specialization, credibility, and coordination (Todorova, 2021; Siamionava et al., 2024).

Specialization refers to the differentiated distribution of expertise among team members (He and Hu, 2021). This cognitive mapping allows individuals to identify and access relevant knowledge sources quickly, thus improving decision-making and problem-solving efficiency (Chen et al., 2023). Credibility reflects the degree of trust that members have in the reliability and validity of each other’s knowledge. High credibility encourages members to adopt and apply others’ inputs without excessive verification costs (Lazar et al., 2022). Coordination captures the extent to which team members can smoothly integrate their efforts, aligning tasks and timing to achieve common objectives (Edwards et al., 2025).

While TMS research mainly focused on professional and organizational teams (Liao et al., 2015; Georgiadou et al., 2024), its principles are highly relevant to undergraduate innovation teams (Kapitsaki et al., 2020). These student-led groups often lack formal hierarchies and depend more on interpersonal trust, shared understanding, and flexible collaboration, making TMS a crucial determinant of their performance.

### 2.2 TMS and innovation performance

Innovation performance reflects a team’s ability to produce novel and useful outcomes (Hughes et al., 2018). A well-developed TMS, as an effective knowledge integration mechanism, can significantly enhance team creativity and innovation performance, which has been demonstrated in many prior studies (Peltokorpi and Hasu, 2016; Fan et al., 2016). For undergraduate innovation teams, where members come from diverse academic backgrounds and must solve complex problems with limited resources, the efficient integration of distributed expertise is vital. TMS provides the structural and relational foundation for such integration (Bachrach et al., 2019).

Specialization refers to the differentiated distribution of knowledge across team members and the shared awareness of “who knows what” within the team (Asim Shahzad et al., 2022). It captures the extent to which members possess distinct domains of expertise and recognize each other’s expertise. In the context of innovation, such cognitive differentiation plays a critical role in enabling teams to integrate knowledge efficiently and creatively (Bachrach et al., 2019; Men et al., 2019).

On the one hand, specialization facilitates effective task allocation and information retrieval. When members are aware of each other’s expertise, domain-specific tasks can be assigned to the most qualified individuals, and members can easily locate and consult the relevant expert when encountering task-related difficulties (Kotlarsky et al., 2015). This reduces redundant information search and duplication of effort, streamlining the problem-solving process and increasing the efficiency of knowledge integration. Moreover, by minimizing cognitive overlap, specialization allows members to focus on deepening their own domain knowledge without having to master all aspects of the team’s knowledge base, thereby reducing cognitive load and increasing overall knowledge quality (Teodoridis et al., 2019).

On the other hand, specialization enhances the diversity of knowledge inputs available to the team. Members with distinct expertise contribute unique perspectives, problem representations, and solution heuristics. Such diversity expands the team’s knowledge base and increases the likelihood of novel combinations, which are a key driver of innovative outcomes (Van Knippenberg and Mell, 2016; Zhou and Li, 2025). Prior research has shown that knowledge diversity arising from specialization enriches the team’s cognitive resources and stimulates creative idea generation (Baruah et al., 2023). In innovation teams composed of members from different academic backgrounds, as is typical in undergraduate innovation projects, this differentiated knowledge structure is particularly valuable for framing complex problems comprehensively and generating novel solutions under resource constraints.

In sum, specialization contributes to innovation performance through two complementary mechanisms: it improves the efficiency of knowledge integration by clarifying “who knows what,” and it enhances the diversity of inputs by pooling differentiated expertise. Both mechanisms increase a team’s capacity to generate novel and useful outcomes. Thus, we have the following assumption:

*H*1a: Specialization of TMS is positively associated with innovation performance in undergraduate innovation teams.

Credibility represents the extent to which team members trust the accuracy and reliability of each other’s expertise (Bachrach et al., 2019). It is a relational dimension of TMS that determines whether knowledge stored in the system will actually be utilized. High credibility reduces doubts and verification costs, thereby fostering smooth and efficient knowledge flows within the team.

First, credibility facilitates effective knowledge utilization by creating an atmosphere of psychological safety and trust. When members have confidence in each other’s expertise, they are more likely to openly share information and incorporate diverse perspectives into collective decision-making (Xu et al., 2023). This trust lowers the perceived risks of relying on others’ knowledge, enabling teams to avoid excessive redundancy and unnecessary re-checking, which often slow down innovation processes (Kmieciak, 2021).

Moreover, credibility enhances collaboration by promoting commitment and reciprocity among team members. Trust in others’ competence encourages individuals to contribute their specialized knowledge willingly, knowing that their input will be valued and appropriately applied (Wang et al., 2023). This mutual respect strengthens intra-team cooperation and facilitates the integration of heterogeneous knowledge, which is essential for generating novel and useful outcomes.

Lastly, credibility accelerates the implementation of innovative ideas by reducing coordination barriers. When members believe in the validity of each other’s expertise, they can align more quickly on solutions and devote more resources to experimentation and problem-solving rather than debating knowledge reliability (Wei et al., 2025). In this way, credibility plays a critical role in transforming potential knowledge diversity into realized innovation performance.

Thus, credibility enhances knowledge sharing, integration, and application by fostering mutual trust and reducing barriers to collaboration. This trust-based mechanism is particularly important in undergraduate innovation teams, which are often temporary and composed of members with limited professional experience. Credibility reduces uncertainty, facilitates open communication, and enables members to quickly coordinate and leverage each other’s diverse but fragmented knowledge. Therefore, the following hypothesis can be drawn:

*H*1b: Credibility of TMS is positively associated with innovation performance in undergraduate innovation teams

Coordination represents the extent to which a team effectively integrates its members’ specialized knowledge and ensures that contributions are aligned with overall team objectives (Todorova, 2021). It is a critical relational and social mechanism within TMS, governing how knowledge is organized, integrated, and applied across tasks.

Initially, effective coordination reduces inefficiencies in knowledge utilization and workflow execution. By clearly defining task dependencies, responsibilities, and timing, coordination prevents duplication of effort, avoids bottlenecks, and ensures that team members can leverage each other’s expertise without conflicts or delays (Xie et al., 2022). This is particularly important in undergraduate innovation teams, where resources are limited and tasks are often complex and interdependent.

Next, coordination facilitates the integration of heterogeneous knowledge by structuring interactions among team members. Well-organized workflows allow specialized insights to be combined systematically, enabling the team to transform diverse knowledge inputs into coherent and innovative solutions (Ruoslahti, 2020). Coordination also supports adaptive learning within teams: as members understand how their contributions fit into the broader process, they can adjust their approaches, refine ideas, and improve overall team performance.

Finally, coordination accelerates the implementation of creative ideas by ensuring that knowledge flows smoothly from conception to execution. When tasks, responsibilities, and dependencies are clearly coordinated, teams can rapidly translate novel insights into actionable outputs, reducing the risk that innovative ideas are lost due to misalignment or delays (Xie et al., 2022).

Altogether, coordination plays a vital role in the performance of undergraduate innovation teams by streamlining workflows, aligning efforts, and ensuring that tasks progress smoothly from planning to execution. Effective coordination helps teams overcome resource limitations, manage interdependencies, and translate creative ideas into actionable outcomes. Accordingly, we can drive the following hypothesis:

*H*1c: Coordination of TMS is positively associated with innovation performance in undergraduate innovation teams.

### 2.3 Moderating effects of credibility and coordination

Specialization reflects the distribution of cognitive resources within a team, allowing members to develop distinct expertise and efficiently identify who holds the relevant knowledge for task execution. However, the value of this cognitive diversity depends on how well members interact socially. Credibility builds mutual trust in each other’s expertise, while coordination aligns actions and workflows to ensure efficient collaboration. When credibility is high and coordination is effective, specialized knowledge is more likely to be shared, integrated, and applied toward innovative outcomes (Vestal and Danneels, 2022, 2024). Thus, credibility and coordination are expected to moderate the relationship between specialization and innovation performance by amplifying or constraining the benefits of cognitive specialization.

We argue that credibility can moderate the relationship between specialization and undergraduate teams’ innovation performance for three reasons. First, credibility reduces the perceived risk and cost associated with utilizing knowledge from others. When team members trust the competence of their peers, they are more willing to seek out and adopt specialized knowledge that is outside their own domain, rather than relying solely on familiar or redundant information (Xu et al., 2023). This reduces unnecessary verification and accelerates knowledge integration, allowing diverse expertise to be effectively leveraged in innovation processes.

Besides, credibility fosters open communication and psychological safety. High trust encourages members to express unconventional ideas and share insights without fear of negative evaluation. In such an environment, specialized knowledge is more likely to be combined with complementary inputs from other members, generating novel recombination that drive breakthrough innovations.

Furthermore, credibility strengthens commitment and reciprocity in knowledge exchange. Members are more likely to contribute their unique expertise proactively when they believe it will be valued and appropriately utilized by the team (Chiu et al., 2016). This mutual trust ensures that specialization translates into actual innovation performance, rather than remaining latent or underutilized. Obviously, we have the following hypothesis:

*H*2a: Credibility strengthens the positive relationship between specialization and innovation performance in undergraduate innovation teams.

In addition to credibility, coordination also serves as a critical contextual factor that determines whether specialization translates into innovation performance. Even when team members possess distinct expertise, the benefits of specialization can be limited if knowledge inputs are not properly aligned with the team’s workflow and project timeline. Coordination governs the sequencing, timing, and integration of specialized knowledge, ensuring that individual contributions complement rather than conflict with one another (Castañer and Oliveira, 2020).

First, effective coordination prevents the isolation of specialized knowledge. When tasks are well-structured and interdependencies are clearly defined, team members can integrate insights from diverse domains in a timely manner, avoiding the risk that valuable expertise remains underutilized or siloed (Xie et al., 2022). This structured integration is essential for enabling novel recombination of knowledge, which is the foundation of innovative outcomes.

Moreover, coordination facilitates adaptive learning within the team. By aligning contributions and clarifying responsibilities, coordination allows members to adjust their approaches based on feedback and ongoing developments. This ensures that specialized knowledge is applied at the right stage of the project, maximizing its impact on the overall innovation process (Mehta et al., 2022).

Finally, coordination enhances efficiency in team workflows. High coordination reduces redundant efforts, prevents bottlenecks, and accelerates the translation of ideas into actionable outputs, thereby strengthening the link between specialization and innovation performance (Huang et al., 2025). In this way, coordination acts as a key moderator that amplifies the positive effects of specialization by ensuring that diverse knowledge inputs are effectively synchronized and implemented. So, we have the subsequent hypothesis:

*H*2b: Coordination strengthens the positive relationship between specialization and innovation performance in undergraduate innovation teams.

**Figure 1:**
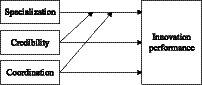
The research model.

Figure 1 presents the integrated framework model that investigates the three dimensions of TMS influencing innovation performance in undergraduate teams.

## 3 Method

### 3.1 Data collection

Undergraduate innovation teams provide valuable context for examining how team-level cognitive and social mechanisms, such as TMS, influence innovation performance in temporary teams. Compared with professional R&D teams, student teams are typically characterized by weaker formal structures and greater reliance on interpersonal trust, self-organized collaboration, and informal coordination. These features make them an ideal setting for investigating the dynamics of knowledge integration in small, temporary, and non-professional teams.

To capture these dynamics, we selected student teams participating in the “Challenge Cup” National Undergraduate Extracurricular Academic and Technological Works Competition as our empirical sample. This competition is widely recognized as one of the most prestigious platforms for undergraduate academic and technological innovation in China. It attracts many interdisciplinary teams formed by students from diverse academic backgrounds and functional roles. These teams work on pre-defined projects under given time limits, which makes them suitable for examining how student groups manage knowledge and generate innovative outcomes.

For practical reasons, we focused on universities in Sichuan Province, China, where we could access to many eligible teams. Based on the competition participation records, 250 teams were identified and were invited to complete a structured survey. To improve the validity of team-level measures, at least two members from each team were asked to fill out the questionnaire independently.

The survey was conducted between February and June 2025, using a combination of paper-based and online questionnaires. Participating teams were drawn from 10 universities across Sichuan. All participants were informed that the data would be used exclusively for academic research and that their responses would remain confidential. The survey followed strict ethical standards, including voluntary participation and anonymous completion, to ensure the authenticity and reliability of the data.

Since the theoretical focus of this study is on team-level mechanisms, the team rather than the individual respondent serves as the unit of analysis. Accordingly, individual responses were aggregated to construct team-level variables. After excluding incomplete and invalid responses, we obtained 852 valid individual questionnaires, which were aggregated into 214 valid team-level observations. This corresponds to a team-level response rate of 85.6% based on the 250 teams initially invited to participate.

### 3.2 Measures

All constructs in this study were measured using multi-item scales adapted from well-established prior research. Responses were recorded on a five-point Likert scale ranging from 1 (“strongly disagree”) to 5 (“strongly agree”), unless otherwise noted. We measured TMS using three dimensions: specialization, credibility, and coordination, following the framework developed by Todorova (2021).

Specialization (5 items) refers to the extent to which team members possess and recognize differentiated areas of expertise. Sample items include: “Each team member has specific expertise,” and “I know which team members are knowledgeable in particular domains.”

Credibility (5 items) captures the level of trust team members place in each other’s knowledge and input. Sample items include: “I trust the knowledge provided by other team members,” and “I have confidence in the information shared by my teammates.”

Coordination (5 items) assesses the quality of interaction and collaboration in the execution of tasks. Sample items include: “Our team works together smoothly and efficiently,” and “We rarely misunderstand each other about how tasks should be done.”

Team innovation performance was evaluated using four subjective items and one objective indicator. The subjective items assessed the novelty, applicability, and efficiency of the team’s outputs (e.g., “Our team often develops novel solutions to problems,” and “Our team is effective in turning creative ideas into applications”), adapted from previous innovation research (Anderson et al., 2014). Additionally, we included a single item to measure objective performance based on the team’s actual award outcome in the competition (1 = no award, 5 = national-level award). These indicators together reflect both perceived and externally validated innovation outcomes.

All individual-level responses were aggregated to the team level by calculating the mean score for each item, as our analysis was conducted at the team level. Table 1 reports all the items and definitions. The options for each item (except for performance_5) were designed in the form of a five-point Likert scale, where 1 = “strongly disagree,” 2 = “basically disagree,” 3 = “hard to say”, 4 = “basically agree”, and 5 = “strongly agree.” Each respondent could only select one score for each item. For item performance_5, objective outcomes were recorded using five categorical levels: (1) no award, (2) faculty-level award, (3) university-level award, (4) provincial-level award, and (5) national-level award.

**Table 1:** Variable items and definitions.

Variables	Items	Definitions
Specialization	specialization_1	Each team member possesses expertise in a specific domain
	specialization_2	I have knowledge in an area that other team members in the project do not possess
	specialization_3	Different team members are responsible for specialized content in different fields
	specialization_4	Delivering the project outcomes requires the expertise of multiple team members
	specialization_5	I know which team members have expertise in specific domains
Credibility	credibility_1	I am willing to accept the professional advice offered by other team members
	credibility_2	I trust that other members’ knowledge about the project is reliable
	credibility_3	I feel confident in the information provided by other members during discussions
	credibility_4	When other members provide information, I tend to verify it myself
	credibility_5	I do not have much confidence in other members’ professional competence
Coordination	coordination_1	Our team collaborates well and works in good harmony
	coordination_2	Our team seldom has misunderstandings about how to carry out specific tasks
	coordination_3	Our team often needs to rework and restart tasks
	coordination_4	Our team completes tasks smoothly and efficiently
	coordination_5	There is a lot of confusion in our team about how we will accomplish tasks
Innovation performance	performance_1	Our team is able to transform innovative ideas into practical applications in a timely manner
	performance_2	Our team often comes up with novel solutions to tasks
	performance_3	Our team works efficiently on innovation-related tasks
	performance_4	Our team produces highly innovative outcomes
	performance_5	Our team’s final achievements in terms of awards
Team size		What is the total number of team members in your team?
Female rate		How many male and female members are there in your team, respectively?
Leadership experience		Does the team leader have previous experience in student associations or project-based collaborations?

To account for potential confounding effects, three control variables at the team level were included. Team size: measured by the total number of team members. Female ratio: measured as the proportion of female members in the team. Leadership experience: whether the team leader held a leadership position in previous experience of student associations or project-based collaboration (1 = yes, 0 = no).

### 3.3 Data analysis

This study employed structural equation modeling (SEM) to test the relationships among the focal constructs. SEM is appropriate for simultaneously examining the measurement model and the structural model while accounting for measurement error. Given that specialization, credibility, coordination, and team innovation performance were treated as latent variables measured by multiple items, SEM was suitable for the present study.

In the empirical analysis, SPSS 29 was used for descriptive statistics, reliability analysis, and exploratory factor analysis (EFA), while AMOS 26 was used for confirmatory factor analysis (CFA) and structural model estimation. Model fit was assessed using multiple indices, including χ^2^/df, RMSEA, CFI, SRMR, and TLI.

## 4 Empirical results

### 4.1 Reliability and validity tests

Before conducting hypothesis testing, the reliability and validity of the measurement scales were examined to ensure the robustness of the results.

Construct validity was examined through convergent validity and structural validity. The Kaiser-Meyer-Olkin (KMO) value was 0.852, which is well above the acceptable threshold of 0.7, indicating sampling adequacy. Bartlett’s test of sphericity was significant (χ^2^ = 2805.902, df = 190, *p* < 0.001), suggesting that the data were suitable for factor analysis (see Table 2).

**Table 2:** Validity analysis of the scales.

KMO		0.852
Bartlett’s test of sphericity	χ^2^ value	2805.902
	Degrees of freedom	190
	*p*-value	0.000

Cronbach’s alpha values were calculated for each construct to assess internal consistency. As shown in Table 3, all constructs demonstrated satisfactory reliability, with Cronbach’s *α* values ranging from 0.704 to 0.812, all exceeding the commonly accepted threshold of 0.70. In terms of convergent validity, all average variance extracted (AVE) values exceeded 0.50, supporting the conclusion that the latent constructs adequately explain the variance of their observed indicators. Composite reliability (CR) values also ranged from 0.846 to 0.870, confirming good internal consistency. Furthermore, standardized factor loadings were all above 0.55, and squared multiple correlations (SMC) supported item reliability. These results indicate that the constructs are measured reliably.

**Table 3:** Reliability analysis of the scales.

Variables	Items	Factor loadings	SMC	CR	AVE	Cronbach’s α
Specialization	specialization_1	0.674	0.454	0.847	0.527	0.812
	specialization_2	0.799	0.638			
	specialization_3	0.740	0.548			
	specialization_4	0.727	0.529			
	specialization_5	0.681	0.464			
Credibility	credibility_1	0.765	0.585	0.870	0.576	0.775
	credibility_2	0.804	0.646			
	credibility_3	0.817	0.667			
	credibility_4	0.818	0.669			
	credibility_5	0.558	0.311			
Coordination	coordination_1	0.736	0.542	0.863	0.557	0.704
	coordination_2	0.744	0.554			
	coordination_3	0.799	0.638			
	coordination_4	0.734	0.539			
	coordination_5	0.717	0.514			
Innovation performance	performance_1	0.774	0.599	0.846	0.525	0.810
	performance_2	0.693	0.480			
	performance_3	0.782	0.612			
	performance_4	0.645	0.416			
	performance_5	0.721	0.520			

In addition, discriminant validity was examined using the Fornell–Larcker criterion. As shown in Table 4, the square roots of the AVE values for all constructs, namely 0.759 for specialization, 0.746 for credibility, 0.725 for coordination, and 0.726 for innovation performance, were all greater than the corresponding inter-construct correlation coefficients. This indicates that each construct shares more variance with its own indicators than with other constructs, thereby demonstrating satisfactory discriminant validity.

**Table 4:** Discriminant validity test results.

Variables	Specialization	Credibility	Coordination	Innovation performance
Specialization	**0.759**			
Credibility	0.600	**0.746**		
Coordination	0.677	0.51	**0.725**	
Innovation Performance	0.619	0.574	0.58	**0.726**

The diagonal numbers in bold are the values of the square root of the average variance extraction (AVE).

### 4.2 Hypotheses testing

#### 4.2.1 The main effects test

Table 5 reports the model fit indices for the main effects model. The value of χ^2^/df was 2.614, which is below the recommended threshold of 3.0, indicating a good fit. The RMSEA value was 0.074, which is within the acceptable range. In addition, the values of CFI and TLI were 0.915 and 0.965, respectively, both exceeding the recommended threshold of 0.90, while the SRMR value was 0.069, below the cutoff value of 0.08. Overall, these results suggest that the proposed structural model achieved an acceptable overall fit to the data.

**Table 5:** Fitness test of the main effects model.

Indicators	χ^2^/df	RMSEA	CFI	SRMR	TLI
Recommended threshold	<3.0	<0.08	>0.9	Close to 0	>0.9
Estimated value	2.614	0.074	0.915	0.069	0.965

**Figure 2:**
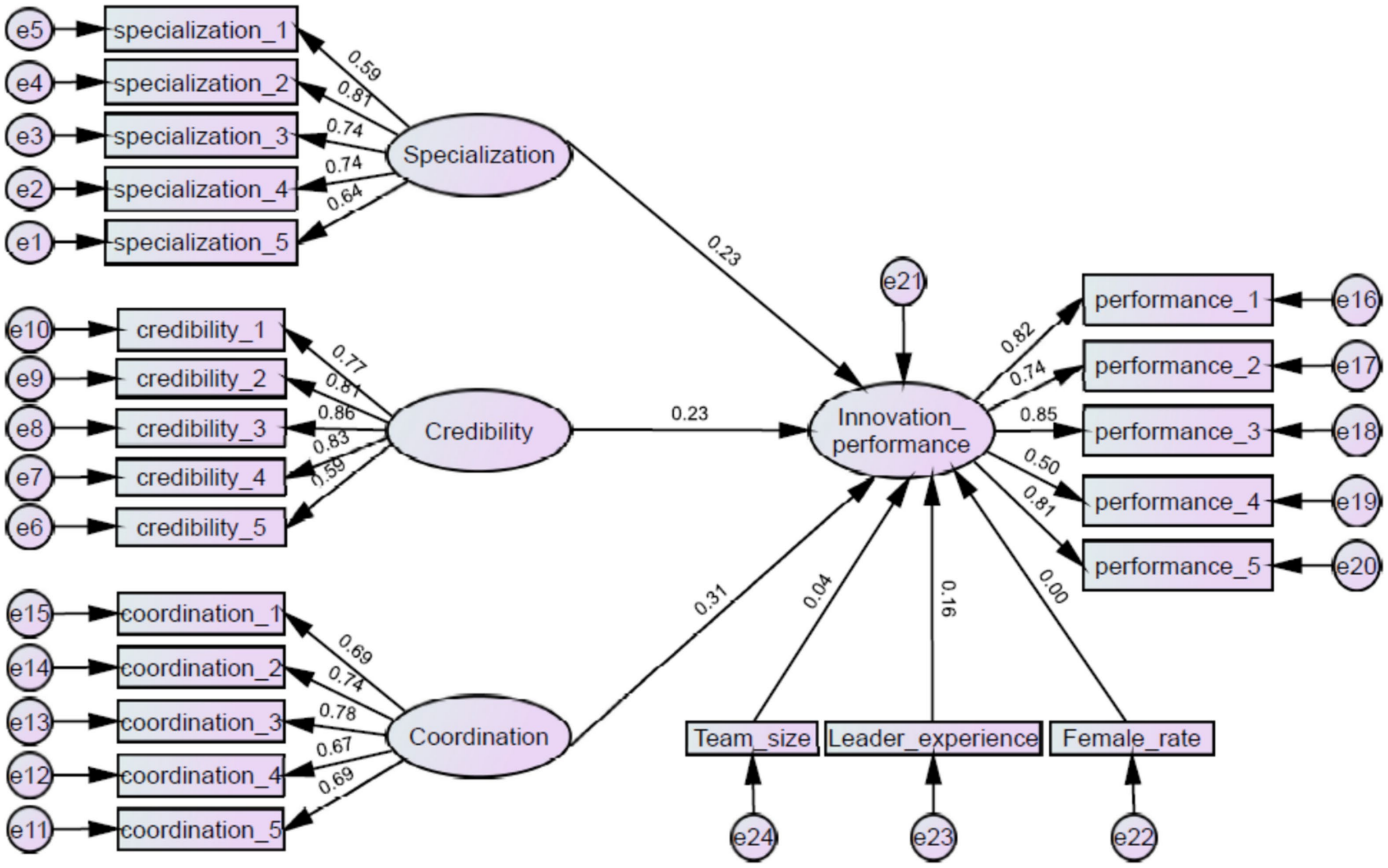
Structural equation model with main effects.

The SEM results are presented in Table 6, and Figure 2 shows the structural equation model with standardized path coefficients. We first examined the direct effects of the three dimensions of TMS on innovation performance in undergraduate innovation teams. As shown in Table 6, specialization has a positive effect on innovation performance (*β* = 0.230, *p* = 0.098), credibility also has a positive effect on innovation performance (*β* = 0.229, *p* = 0.074), and coordination has a significantly positive effect on innovation performance (*β* = 0.309, *p* = 0.004). With regard to hypothesis testing, the path from specialization to innovation performance is positive and significant at the 10% level, supporting H1a. Likewise, the path from credibility to innovation performance is positive and significant at the 10% level, supporting H1b. The path from coordination to innovation performance is positive and significant at the 1% level, providing strong support for H1c.

**Table 6:** Results of main effects.

Hypotheses	Path	Standard regression weights	*p*-value
H1a	Specialization→Innovation performance	0.230*	0.098
H1b	Credibility→Innovation performance	0.229*	0.074
H1c	Coordination→Innovation performance	0.309***	0.004
	Team_size→Innovation performance	0.042	0.971
	Female_rate→Innovation performance	0.002	0.437
	Leader_experience→Innovation performance	0.156***	0.005

***p < 0.01, **p < 0.05, *p < 0.1.

Regarding the control variables, team size does not have a significant effect on innovation performance (*β* = 0.042, *p* = 0.971), and female proportion is also not significantly related to innovation performance (*β* = 0.002, *p* = 0.437). In contrast, leadership experience has a significantly positive effect on innovation performance (*β* = 0.156, *p* = 0.005), suggesting that teams led by members with prior leadership experience tend to achieve better innovation outcomes.

Taken together, the findings indicate that the three dimensions of TMS are all positively associated with innovation performance in undergraduate innovation teams.

#### 4.2.2 The moderating effects test

To test the moderating effects between latent constructs, this study adopted the product indicator approach in AMOS. Following prior research, the indicators of the focal latent variables were first mean-centered to reduce multicollinearity, and then matched and multiplied pairwise to construct the product indicators for the latent interaction terms. These interaction terms were subsequently incorporated into the structural equation model for hypothesis testing.

Using the maximum likelihood estimation method, the structural model with the interaction terms demonstrated a good overall fit to the data. Specifically, the model fit indices were as follows: χ^2^/df = 1.555, RMSEA = 0.015, CFI = 0.951, TLI = 0.947, and SRMR = 0.019. These values indicate that the moderating effects model was appropriate for subsequent hypothesis testing.

**Figure 3:**
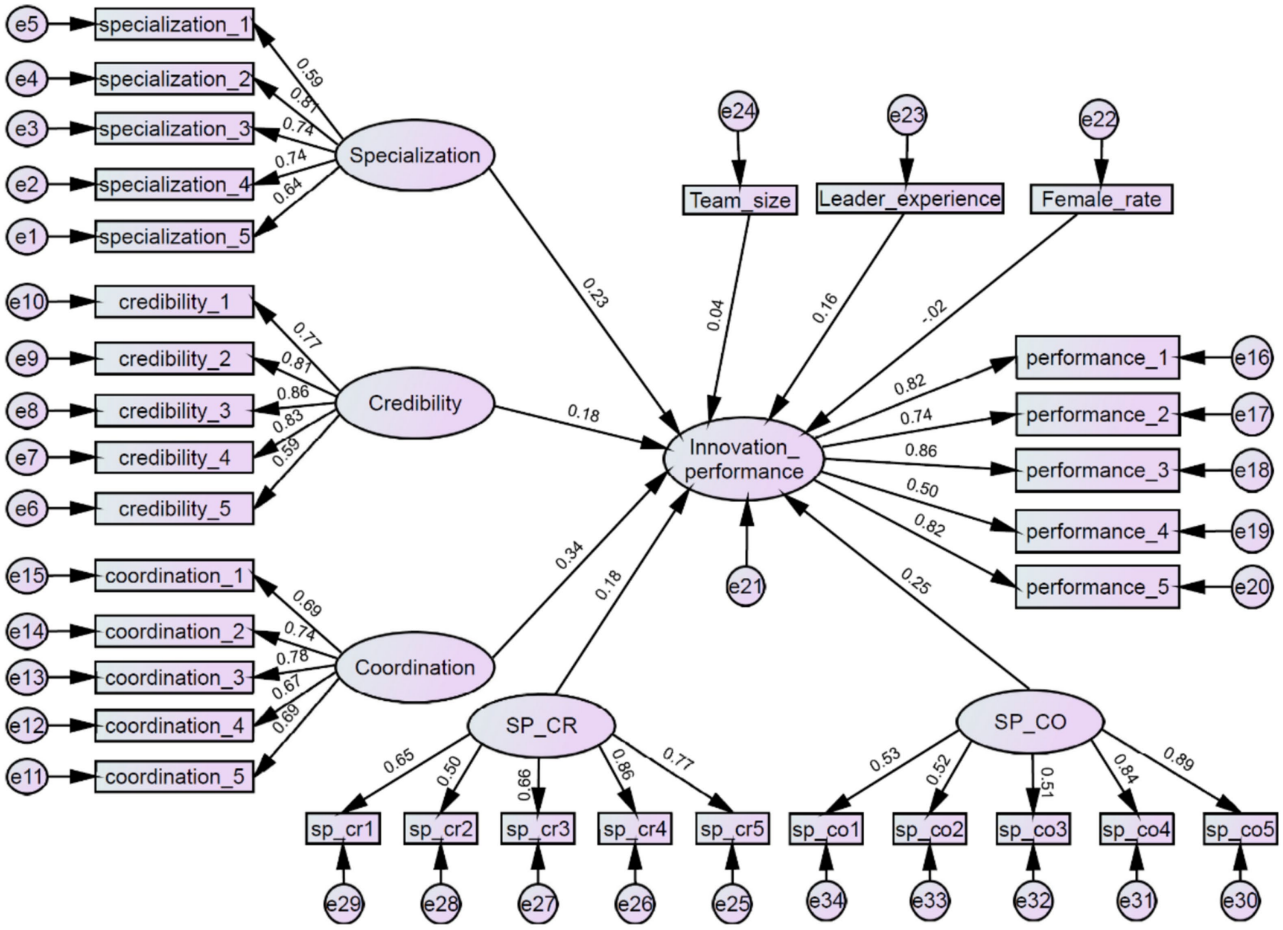
Structural equation model with moderating effects.

The results of the moderating effects analysis are presented in Table 7, and Figure 3 shows the structural equation model with moderating effects. As shown in Table 7, the interaction term between specialization and credibility has a significantly positive effect on innovation performance (*β* = 0.184, *p* = 0.009), supporting H2a. This result suggests that credibility positively moderates the relationship between specialization and innovation performance. Similarly, the interaction term between specialization and coordination is also significantly and positively associated with innovation performance (*β* = 0.250, *p* = 0.002), providing support for H2b. This finding indicates that coordination strengthens the positive relationship between specialization and innovation performance.

**Table 7:** Results of moderating effects.

Hypotheses	Path	Estimate	*p*-value
H2a	Specialization*Credibility→Innovation performance	0.184***	0.009
H2b	Specialization*Coordination→Innovation performance	0.250***	0.002
	Specialization→Innovation performance	0.233*	0.084
	Credibility→Innovation performance	0.179	0.145
	Coordination→Innovation performance	0.337***	0.001
	Team size→Innovation performance	0.045	0.392
	Female rate→Innovation performance	−0.015	0.774
	Leader experience→Innovation performance	0.16***	0.003

***p < 0.01, **p < 0.05, *p < 0.1.

**Figure 4:**
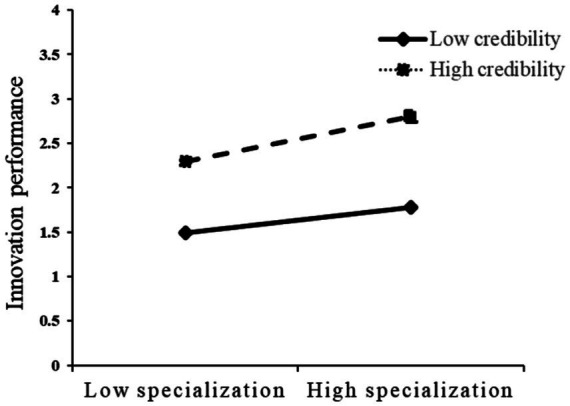
The moderating effect of credibility.

**Figure 5:**
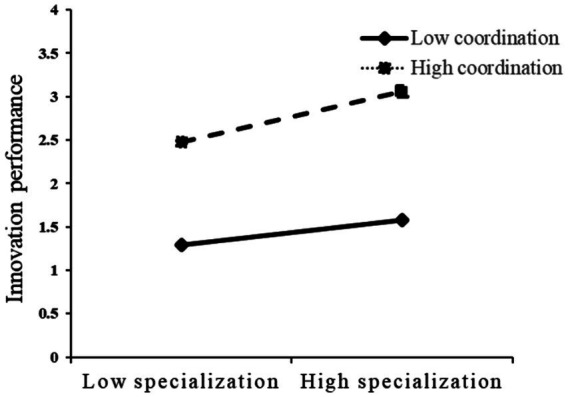
The moderating effect of coordination.

To further illustrate the moderating effects, Figures 4, 5 plot the simple slopes of the moderating effects. As shown in the figures, the positive relationship between specialization and innovation performance becomes stronger when credibility and coordination are high rather than low, which provides additional visual support for H2a and H2b.

### 4.3 Robustness

To further assess the robustness of our results, we replaced the dependent variable with a more objective indicator of team innovation performance, namely the team’s award-winning status, measured by the fifth item of the innovation performance scale. Using this alternative measure, denoted as Performance_ob, we re-estimated both the main-effects model and the moderating-effects model. As reported in Table 8, the results remain substantively unchanged: specialization, credibility, and coordination continue to exert positive effects on innovation performance, and the moderating roles of credibility and coordination in the relationship between specialization and innovation performance remain significant.

**Table 8:** Results of robustness test.

Hypotheses	Path	Estimate	*p*-value
H1a	Specialization→Performance_ob	0.281**	0.044
H1b	Credibility→Performance_ob	0.255*	0.052
H1c	Coordination→Performance_ob	0.148**	0.041
	Team_size→Performance_ob	0.006	0.917
	Female_rate→Performance_ob	0.094*	0.094
	Leader_experience→Performance_ob	0.184***	0.001
H2a	Specialization*Credibility→Performance_ob	0.175**	0.015
H2b	Specialization*Coordination→Performance_ob	0.173**	0.024
	Specialization→Performance_ob	0.288**	0.036
	Credibility→Performance_ob	0.203	0.114
	Coordination→Performance_ob	0.084***	0.008
	Team size→Performance_ob	0.002	0.978
	Female rate→Performance_ob	0.087	0.116
	Leader experience→Performance_ob	0.185***	0.000

***p < 0.01, **p < 0.05, *p < 0.1.

## 5 Discussion

Although TMS have attracted increasing scholarly attention in organizational and educational contexts, its role in temporary undergraduate innovation teams has received limited attention. Building on prior research on TMS and team innovation, this study developed and tested a model focusing on the effects of three core TMS dimensions (specialization, credibility, and coordination) on innovation performance in undergraduate teams. Furthermore, recognizing that these dimensions may not function independently, we examined whether credibility and coordination moderate the relationship between specialization and innovation performance. Using survey data from undergraduate teams participating in innovation and entrepreneurship programs, our findings indicate that all three dimensions significantly enhance innovation performance, and that the positive effect of specialization is amplified when teams demonstrate higher levels of credibility and coordination. These findings suggest that TMS in temporary undergraduate innovation teams should be understood not only as a set of differentiated dimensions, but also as a team-level mechanism in which cognitive and social-interaction elements work together to support innovation.

### 5.1 Theoretical implications

This study advances the literature on TMS and team innovation in several ways. First, this study extends TMS research to a type of team that has received limited attention in prior work, namely temporary undergraduate innovation teams. Existing TMS studies have mainly focused on organizational teams such as R&D teams, project teams, or virtual collaboration groups in corporate settings (Bachrach et al., 2019). By contrast, undergraduate innovation teams are typically temporary, weakly formalized, and characterized by short interaction histories and limited procedural support. In such settings, team members cannot rely heavily on established routines, formal hierarchy, or stable role structures to integrate distributed knowledge. Our findings therefore extend the relevance of TMS theory to temporary teams and show that TMS remains an important mechanism for coordinating expertise and supporting innovation even when formal organizational arrangements are limited.

Second, this study advances the broader TMS literature by showing that the three dimensions of TMS should be understood not only as functionally distinct, but also as interrelated. Prior research has increasingly moved beyond treating TMS as a single aggregate construct and has begun to examine its dimensions separately. Building on this stream of research, our study suggests that dimensional TMS should not be understood merely as the decomposition of a higher-order construct into parallel components. Rather, the three dimensions represent related aspects of team knowledge integration. In particular, our results indicate that specialization, as a cognitive dimension that maps distributed expertise, is not sufficient by itself; its positive effect on innovation performance depends partly on whether members trust one another’s expertise and can coordinate their contributions effectively. This finding contributes to TMS theory by showing that social-interaction dimensions help shape the extent to which the cognitive value of specialization is realized in practice.

Third, this study contributes to the innovation and education literature by clarifying how distributed expertise is translated into innovation performance in interdisciplinary student teams. Research on team innovation has long emphasized the value of knowledge diversity, yet diversity alone does not automatically generate innovative outcomes. Our findings show that in undergraduate innovation teams, the innovative potential created by differentiated expertise is more likely to be realized when teams also develop mutual credibility and effective coordination. This provides a more fine-grained explanation of how team-level innovation emerges in educational settings. In this sense, the study contributes not only to the student-team context, but also to the broader innovation literature by demonstrating that innovation performance depends not simply on the presence of distributed knowledge, but on the interaction between cognitive differentiation and social integration mechanisms. At the same time, it enriches innovation and entrepreneurship education research by explaining why some interdisciplinary student teams are better able than others to transform academic diversity into innovative outcomes.

### 5.2 Managerial implications

The findings of this study are particularly relevant for universities, for example in Sichuan Province, where innovation and entrepreneurship education has been increasingly promoted through competitions, project-based learning, and interdisciplinary training activities. Beyond improving competition outcomes, these activities can also guide students in a positive direction by strengthening their sense of responsibility, collaboration skills, communication ability, and problem-solving capacity. From this perspective, undergraduate innovation teams should be viewed not only as vehicles for project completion, but also as important platforms for student development and talent cultivation.

First, fostering clear specialization among team members is essential. Team formation processes should emphasize diversity in disciplinary backgrounds and explicitly clarify individual expertise areas, enabling members to quickly locate relevant knowledge when needed. For example, in a university innovation team in Sichuan, engineering students may be responsible for technical development, business students for market analysis and feasibility assessment, and design students for user-interface or presentation design. When each member’s expertise is clearly identified at the beginning of the project, the team can reduce confusion, improve communication efficiency, and make better use of interdisciplinary knowledge. In future practice, universities may formalize this process by requiring teams to prepare an “expertise map” during the project launch stage.

Second, building credibility should be a central objective in early project stages. Facilitators can implement trust-building exercises, encourage open communication, and create opportunities for members to demonstrate competence in their respective domains. Such interventions can strengthen the willingness of members to rely on each other’s knowledge, thereby enhancing the benefits of specialization.

Third, enhancing coordination mechanisms is vital for converting cognitive diversity into practical outcomes. Structured project management tools (e.g., shared timelines, task boards, or workflow software) can help synchronize contributions and reduce the risk of delays or redundancy. Instructors and mentors can provide guidance on setting clear milestones and aligning deliverables across different roles.

Finally, given the moderating effects identified, managerial attention should be placed not only on building individual TMS dimensions but also on ensuring their alignment. High specialization without sufficient credibility or coordination may fail to improve innovation performance, whereas balanced development of all three dimensions is more likely to maximize team effectiveness.

### 5.3 Limitation and future research directions

This study has several limitations that should be noted. First, the data were collected from undergraduate innovation teams in China, which may limit the generalizability of the findings to other cultural or institutional settings. Second, the cross-sectional and self-reported nature of the data restricts causal inference and may introduce perceptual bias. Future research could adopt longitudinal or experimental designs, incorporate objective performance measures, and examine additional moderators such as psychological safety, leadership style, or digital collaboration tools to provide a more comprehensive understanding of how TMS influences innovation performance.

## Data Availability

The raw data supporting the conclusions of this article will be made available by the authors, without undue reservation.
